# A Case of Paraganglioma-Induced Adrenergic Shock

**DOI:** 10.7759/cureus.26925

**Published:** 2022-07-16

**Authors:** Mariana S Santos, Maria Margarida Teixeira, Abel Alves, Bernardo Dias Pereira, Manuela Henriques

**Affiliations:** 1 Department of Intensive Care Medicine, Hospital do Divino Espírito Santo de Ponta Delgada, Ponta Delgada, PRT; 2 Department of Endocrinology and Nutrition, Hospital do Divino Espírito Santo de Ponta Delgada, Ponta Delgada, PRT

**Keywords:** prognosis, diagnosis, adrenergic shock, life-threatening, paraganglioma

## Abstract

Paragangliomas (PGLs) are rare neuroendocrine tumors that, when functional, can release excessive catecholamines, causing health conditions ranging from asymptomatic arterial hypertension to life-threatening arrhythmias and cardiogenic shock. Early diagnosis of functional PGLs is extremely important as timely treatment can be curative and prevent vascular sequelae. We describe the clinical case of a 30-year-old woman with arterial hypertension under study, who was presented to the emergency department with a hypertensive crisis that progressed to adrenergic shock, in the context of a functional PGL.

## Introduction

Paraganglioma (PGL) is a neuroendocrine tumor of chromaffin cells originating from the sympathetic and parasympathetic ganglia located in the nervous plexuses of the autonomic nervous system, from the base of the skull to the pelvis. It represents 15%-20% of chromaffin cell tumors, which are originated mostly in the medulla of the adrenal gland (pheochromocytoma) [1]. PGL and pheochromocytoma have an annual incidence of 3-8 per 1,000,000 individuals and represent 0.05%-0.1% of cases of resistant hypertension (HT) [2]. They are usually benign and unifocal tumors, and have a genetic origin in 30%-40% of cases. In this context, multifocality and a greater propensity to malignancy (according to the mutated gene) are often observed [3]. PGL affects both genders and has a peak incidence at 40-50 years of age, but on average, it develops a decade earlier when associated with hereditary syndromes [3,4].

Parasympathetic PGLs, usually located in the head and neck, are nonfunctional in 95% of cases, while sympathetic PGLs, usually located in the thorax and abdomen, synthesize, store and secrete excess of catecholamines [3]. The clinical manifestations of functional PGLs can range from asymptomatic isolated HT to vascular complications usually related to massive secretion of catecholamines and its consequent hypertensive crisis. Cardiovascular complications of PGL occur in 25%-33% of individuals and can be arrhythmias, myocarditis, dilated or obstructive cardiomyopathy or Takotsubo syndrome [5].

According to the literature, the existence of shock is a rare manifestation of the diagnosis of pheochromocytoma. It affects 2% as the initial manifestation [6,7]. The exact pathophysiology of shock is still not fully understood [8]. Here, we report a case of a 30-year-old woman, with a history of HT under investigation for secondary causes, who was admitted to the emergency department due to shock of unknown etiology that was identified as adrenergic shock caused by an abdominal PGL.

## Case presentation

A 30-year-old female was brought to the emergency department with general malaise, palpitations, retrosternal discomfort, diaphoresis, and vomits of food content associated with HT. She had a previous history of thyroid nodules and HT diagnosed two years ago. There was no family history of paraglangioma/pheochromocytoma. The patient was medicated with perindopril 5 mg and, in retrospect, bisoprolol 5 mg had recently been suspended in an endocrinology consultation for suspected pheochromocytoma, in the context of typical paroxysmal symptoms occurring weekly, associated with HT. Her family history was irrelevant. On physical examination, she was restless and sweaty, with severe HT (>180/110 mmHg in all limbs), sinus tachycardia (110-150 bpm), signs of poor peripheral perfusion and cold extremities, and apyrexia; the Glasgow Coma Scale score was 15. Biochemically, she had metabolic acidemia and hyperlactacidemia of 5 mmol/L (reference value <2 mmol/L), leukocytosis with neutrophilia and a low C-reactive protein level, acute renal failure, a mixed pattern of elevated liver enzymes, a high pro-B-type natriuretic peptide (pro-BNP) level and a slight increase in cardiac necrosis markers (Table 1).

**Table 1:** Biochemical surveys performed in the emergency room ALT: alanine aminotransaminase; AST: aspartate aminotransaminase; GGT: gamma glutamyl transferase; pro-BNP: pro-B-type natriuretic peptide

Blood analysis	Results	Reference value
Hemoglobin (g/dL)	14.0	12-15.5
Leukocytes (x10^3^ uL)	20.7	4-11.5
Relative neutrophils (%)	84.4	50-70
Platelets (x10^3^ uL)	413	150-400
C-reactive proteins (mg/dL)	0.15	0.0-0.5
Procalcitonin (ng/mL)	7.87	<0.05
Urea (mg/dL)	41	10-50
Creatinine (mg/dL)	2.1	0.7-1.3
ALT (U/L)	391	10-40
AST (U/L)	298	<34
GGT (U/L)	581	<73
Alkaline phosphatase (U/L)	151	46-116
Total bilirubin (mg/dL)	0.75	0.3-1.2
Direct bilirubin (mg/dL)	0.36	<0.31
Pro-BNP (pg/mL)	1209	<450
Troponin I (ug/L)	3.47	<0.045
Phosphorus (mg/dL)	3.9	2.5-4.9
Calcium total /mg/dL)	9.2	8.3-10.6
Calcium (mg/dL)	9.1	8.5-10.1
Albumin (g/dL)	4.1	3.4-5

**Figure 1:**
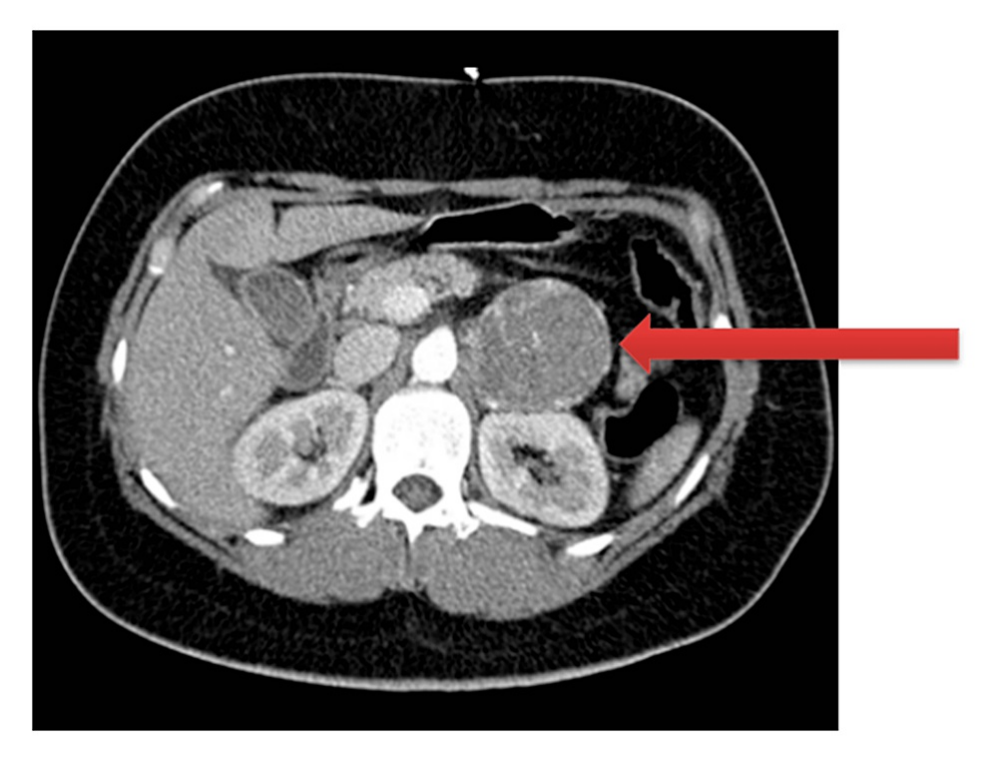
Computed tomography scan of the abdomen, showing the paraganglioma (red arrow)

The electrocardiogram on admission showed sinus tachycardia and several supraventricular extrasystoles. A transthoracic echocardiogram was performed and showed concentric left ventricular hypertrophy with ejection fraction (LVEF) moderately depressed (39%-41%) by diffuse hypocontractility. Antihypertensive therapy was instituted with nifedipine 30 mg, isosorbide dinitrate (2 mg bolus followed by an infusion of 4 mg/h) and clonidine 0.15 mg. Serial blood gas analysis revealed a decrease in the lactate level after fluid therapy, but type 1 respiratory failure ensued, probably due to hypervolemia. A full-body computed tomography (CT) scan was performed that revealed a left retroperitoneal mass, lateral to the adrenal gland, measuring 73 x 56 x 57 mm, with marked contrast enhancement, suspected of a PGL (Figure 1).

The diagnosis of mixed shock (adrenergic and cardiogenic shock) with hypoxemic respiratory failure secondary to hypervolemia was assumed, eventually induced by the PGL. She was admitted to the intensive care unit for further management. She required non-invasive ventilation and continuous infusion of low-dose furosemide, without the need for antihypertensive drugs, with significant improvement. A transthoracic echocardiogram was performed on the ninth day of hospitalization, which revealed improvement in biventricular systolic function (LVEF: 55%), mild pericardial effusion (without hemodynamic compromise), and concentric left ventricular hypertrophy (LVH). The biochemical endocrine study revealed a marked elevation in plasma and 24-hour urinary metanephrines (Table 2).

**Table 2:** Endocrine biochemical surveys

Urine and blood analysis		Results	Reference value
Urinary fractionated metanephrines (ug/24 h)		6668	<785
Urinary fractionated catecholamine/24 h	Adrenaline (ug/24 h)	57	<18
	Noradrenaline (ug/24 h)	155	<76
	Dopamida (ug/24 h)	330	<390
Plasma fractionated metanephrines/24 h	Metanephrines (pg/mL/24 h)	838	<64
	Normetanephrines (pg/mL/24 h)	1296	<196
Plasma fractionated catecholamine/24 h	Adrenaline (pg/mL/24 h)	432	<20.60
	Noradrenaline (pg/mL/24 h)	6074	10-150
	Dopamine (pg/mL/24 h)	300	<300
Parathyroid hormone (pg/mL)		78.1	11.1-79.5
Thyroid-stimulating hormone (uUl/mL)		0.137	0.35-4.97
Thyroid hormone (T3) (pg/mL)		2.68	1.71-3.71
Thyroid hormone (T4) (ng/dL)		0.88	0.7-1.48

She was transferred to the endocrinology ward on the seventh day of hospitalization. At the 12th day of hospital admission, the patient had a recurrent adrenergic shock presenting with hypertension, excessive sweating, and cold extremities. After a multidisciplinary discussion, it was decided to proceed with the surgical removal of the adrenal mass. Due to the lack of resources to perform scintigraphy with metaiodobenzylguanidine (MIBG scan), an abdominal magnetic resonance imaging (MRI) scan was performed to possibly approach surgery and alpha adrenergic blockade was initiated prior to the surgery.

**Figure 2:**
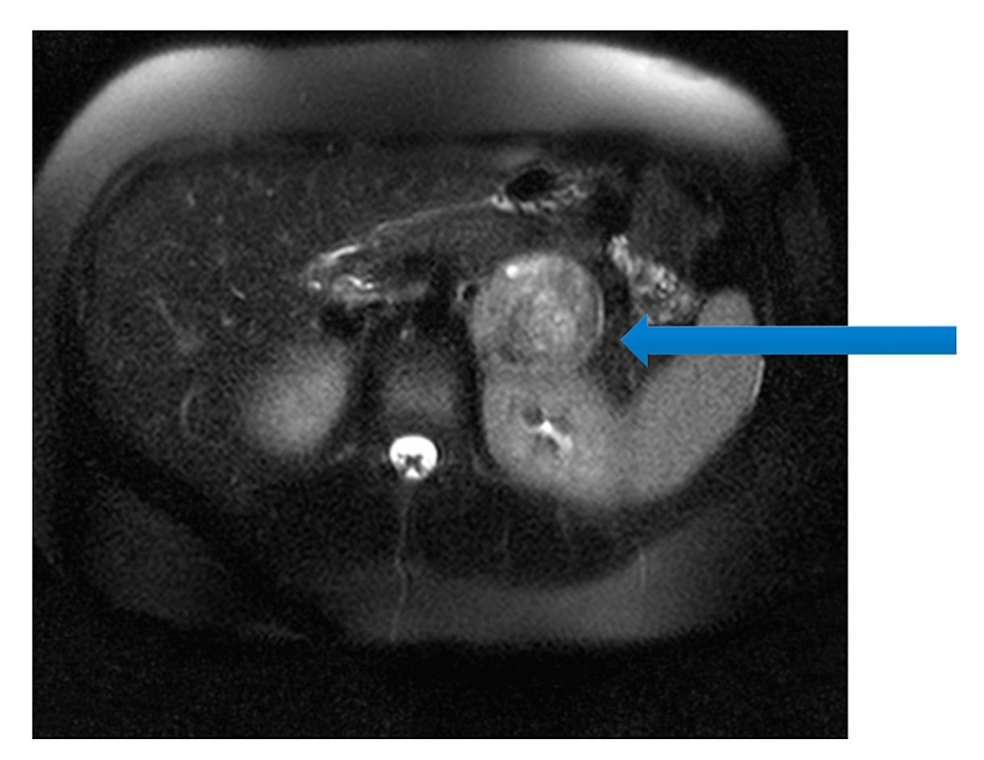
Magnetic resonance imaging of the abdomen in T2, showing the left mass corresponding to the paraganglioma (blue arrow)

MRI showed a left paramedian retroperitoneal mass, hyperintense on T2-weighted images, with no invasion of adjacent structures, measuring 50 x 51 x 64 mm (Figure 2).

She was started on phenoxybenzamine 10 mg each 12 hours, titrated up to every 8 hours. She underwent laparoscopic excision of the mass on the 22nd day of hospitalization. Histologically, the mass consisted of cells with granular and basophilic cytoplasm, in solid nests delimited by vascularized connective tissue, without necrosis, with positive immunostaining for chromogranin A and synaptophysin, a Ki-67 index <1% and <2 mitoses/HPF. The mass was surrounded by a fibrous capsule, with no signs of invasion. Genetic screening performed by the next-generation sequencing panel (TruSight® Cancer Gene Set; Illumina, San Diego, CA) including all coding exons and splicing regions of RET, VHL, SDHA/B/C/D/AF2, TMEM127, MAX, and FH genes, and by multiplex ligation-dependent probe amplification for large deletions/duplications of VHL and SDHB/C/D/AF1/AF2 was negative. The patient was discharged on the 29th day of hospitalization, asymptomatic and normotensive without antihypertensive therapy. She maintains her annual long-term clinical and biochemical follow-up (24-hour plasma/urinary metanephrines).

## Discussion

Here, we report a case of a 30-year-old woman diagnosed with mixed shock (adrenergic and cardiogenic) with concentric LVH, in the context of excess catecholamines secreted by a functional PGL. The most common site where these tumors develop is the abdomen, generally in the para-aortic and perirenal spaces, but they may also be located in the thoracic and pelvic regions [4,9,10]. A functional PGL presents clinically with paradoxical or sustained HT associated with tachycardia, headache, diaphoresis, anxiety, and episodic palpitations [4,11]. According to the literature, the existence of shock is a rare manifestation of pheochromocytoma diagnosis. It affects 2% as an initial manifestation and its exact pathophysiology is not yet fully understood [6-8]. Laboratory diagnosis is made with the measurement of metanephrines in the plasma and urine, although the former is nowadays preferred [12].

MRI is superior to CT as a screening imaging method for PGLs, although CT may be preferred in certain circumstances for head and neck tumors (superior spatial resolution, less motion artifacts, precise determination of extension into the bone) [13]. Both imaging methods are thus thought as complementary for staging and to ascertain multifocality. Functional imaging is used in the management of a PGL to localize the primary tumor, define the tumor burden of a metastatic PGL that may be missed on CT or MRI surveys, and characterize the metabolic activity of the PGL for therapeutic purposes. The accuracy of available functional imaging tools is highly dependent on the specific mutation of PGL-associated genes, and recommendations regarding the best functional imaging according to the patient genotype have been published in recent years [14].

According to the literature, the cardiovascular responses to catecholamine depend mostly on which one that is produced by the adrenal mass. The acute release of norepinephrine and epinephrine increases heart rate, systemic vascular resistance, and myocardial contractility, and reduces venous compliance. The excessive adrenergic stimulation by catecholamines results in severe vasoconstriction and coronary vasospasm, myocardial ischemia, and subsequently necrosis. Consequently, this can lead to severe acute (acute coronary syndromes and Takotsubo cardiomyopathy) or chronic (hypertensive heart disease, catecholamine-induced cardiomyopathy) cardiovascular complications [15].

The best treatment in the acute phase is the administration of alpha- and beta-adrenergic blocking drugs. If the condition persists, surgical resection of the PGL should be performed [15]. Due to the new recurrence of an adrenergic crisis during hospitalization, the patient was proposed for surgical treatment. According to the literature, preoperative preparation is recommended to minimize surgical complications and is indicated in functional PGLs, given the increased risk of perioperative hypertensive crisis, arrhythmias, severe hypotension, or hypovolemic shock. The main goals of preoperative preparation are the normalization of blood pressure and heart rate and correction of hypovolemia with alpha-adrenergic blockade (preferably phenoxybenzamine) and beta-adrenergic blockade, if needed (e.g., heart rate >90 bpm), that should be introduced at least 7-14 days before surgery [5,16].

The long-term prognosis of a sporadic, solitary tumour is excellent, although HT may persist in 50% of patients [17]. The recurrence rate is 17%, being more common in cases of extra-adrenal location (33%) and familial association (33%). All patients should be followed annually for at least 10 years. In cases of paraganglioma or in hereditary situations, they should be followed indefinitely [17]. According to the literature, recurrence of PGLs may occur up to 20 years after initial presentation, so follow-up should be lifelong and include clinical, biochemical, and imaging evaluation [4]. Postoperative follow-up with tests for annual plasma or urinary metanephrines and chromogramin A is indicated in all patients with functional PGLs.

## Conclusions

Adrenergic shock induced by a functional PGL is a rare diagnosis of uncertain pathophysiology. Early diagnosis is very important, requiring a high index of clinical suspicion, and surgical treatment is potentially curative. This case also highlights the importance of alpha-adrenergic blockade in the preoperative preparation. The prognosis is globally good, but high recurrence rates have been seen, and thus lifelong follow-up is recommended.
